# Validated RP-HPLC Method for Simultaneous Quantitation of Losartan Potassium and Metolazone in Bulk Drug and Formulation

**DOI:** 10.3797/scipharm.1105-13

**Published:** 2011-06-26

**Authors:** Ramkumar Dubey, Vidhya K. Bhusari, Sunil R. Dhaneshwar

**Affiliations:** Department of Pharmaceutical Chemistry, Bharati Vidyapeeth University, Poona College of Pharmacy, Pune, Maharashtra, 411038, India

**Keywords:** Losartan potassium, Metolazone, HPLC, Validation, Metoz^©^

## Abstract

A HPLC method has been described for simultaneous determination of Losartan potassium and Metolazone in formulation. This method is based on a HPLC separation of the two drugs on the Thermo Hypersil BDS–C_18_ (250 mm × 4.6 mm, 5.0 μm) with isocratic conditions and a simple mobile phase containing acetonitrile:water (60:40) at a flow rate of 0.8 mL/min using UV detection at 237 nm. This method has been applied to a marketed formulation without interference of excipients. The linear regression analysis data for the calibration plots showed a good linear relationship over the concentration range of 2–12 μg/mL for Losartan potassium and 0.2–1.2 μg/mL for Metolazone, respectively. The method was validated for precision, robustness and recovery. Statistical analysis showed that the method is repeatable and selective for the estimation of Losartan potassium and Metolazone.

## Introduction

**Fig. 1.**
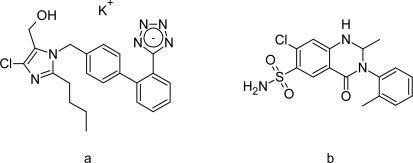
Structures of Losartan potassium (a) and Metolazone (b)

Losartan potassium is chemically potassium 5-(4′-{[2-butyl-4-chloro-5-(hydroxymethyl)-1*H*-imidazol-1-yl]methyl}biphenyl-2-yl)tetrazol-1-ide (Figure 1a) [1]. It is a strong non-peptide antihypertensive agent, which exerts its action by specific blocking of angiotensin II receptors. It has a gradual, long-lasting effect as an antihypertensive.

Metolazone is chemically 7-chloro-2-methyl-3-(2-methylphenyl)-4-oxo-1,2,3,4-tetrahydro-quinazoline-6-sulfonamide [2, 3] (Figure 1b). Metolazone is an oral diuretic drug, commonly classified with the thiazide diuretics. It is primarily used to treat congestive heart failure and high blood pressure. Metolazone indirectly decreases the amount of water reabsorbed into the bloodstream by the kidney, so that blood volume decreases and urine volume increases. This lowers blood pressure and prevents excess fluid accumulation in heart failure.

Hence, the combination of Losartan potassium and Metolazone (extended release), such as in the marketed product Metoz^©^, complements each other and provides an additive effect on blood pressure control, which is sustained for at least 24 hours.

Literature review reveals that methods have been reported for analysis of Losartan potassium and Metolazone, HPLC method for determination of Losartan potassium in combination with other drugs [4–6] and few bioanalytical methods are also reported [7–10]. RP-HPLC method for determination of Metolazone [11] and few bioanalytical methods are also reported [12–14].

To date, there have been no published reports about the simultaneous quantitation of Losartan potassium and Metolazone by HPLC in bulk drug and in tablet dosage form. This present study reports for the first time simultaneous quantitation of Losartan potassium and Metolazone by RP-HPLC in bulk drug and in tablet dosage form. The proposed method is validated as per ICH guidelines [15–17].

## Experimental

### Materials

Centaur Pharmaceuticals Pvt. Ltd. Ambarnath, India, kindly supplied pure drug sample of Losartan potassium as a gift sample of Batch No.: LTP/100/002 and Metolazone of Batch No.: 2010360IP. It was used without further purification and certified to contain 99.60 % (w/w) for Losartan potassium and 99.70 % (w/w) for Metolazone on dried basis. All chemicals and reagents used were of HPLC grade and were purchased from Merck Chemicals, India.

### Instrumentation

The HPLC system consisted of a Pump (model Jasco PU 2080), Intelligent LC pump with sampler programmed at 20 μL capacity per injection was used. The detector consisted of UV/VIS (Jasco UV 2075) model operated at a wavelength of 237 nm. Data was integrated using Jasco Borwin version 1.5, LC-Net II/ADC system. The column used was a Thermo Hypersil BDS–C_18_ (250 mm × 4.6 mm, 5.0 μm) from Germany.

### Preparation of Standard Stock Solutions

Standard stock solution of concentration 100 μg/mL of Losartan potassium and 10 μg/mL of Metolazone was prepared using acetonitrile. From the standard stock solution, the mixed standard solutions were prepared using mobile phase (acetonitrile:water in the ratio 60:40 v/v) to contain 20 μg/mL of Losartan potassium and 2 μg/mL of Metolazone. The stock solution was stored at 2–8 °C protected from light.

### Optimization of HPLC Method

**Fig. 2.**
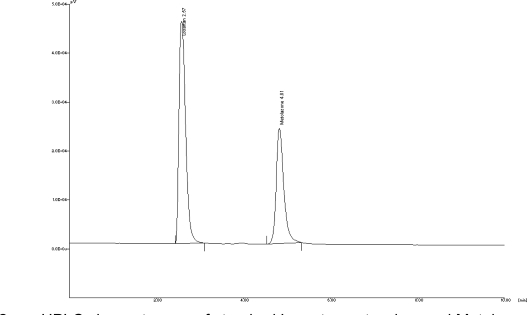
HPLC chromatogram of standard Losartan potassium and Metolazone (20 μg/mL and 2 μg/mL)

The HPLC procedure was optimized with a view to develop a simultaneous assay method for Losartan potassium and Metolazone respectively. The mixed standard stock solution (20 μg/mL of Losartan potassium and 2 μg/mL of Metolazone) was injected in HPLC. For HPLC method optimization different ratios of methanol and water were tried but it was found that the peaks got distorted when methanol was used. Hence, methanol was replaced with acetonitrile. Different ratios of acetonitrile and water were tried. It was found that acetonitrile: water in the ratio 60:40 v/v at flow rate 1.0 mL/min gave good peak shape but it was found that the drugs eluted in dead volume. Hence the flow rate was reduced to 0.8 mL/min which gave acceptable retention time (t_R_) of 2.57 and 4.81, the plate count was found to be 4950 and 5548 and good resolution for Losartan potassium and Metolazone, respectively (Figure 2).

### Validation of the method

Validation of the optimized HPLC method was carried out with respect to:

#### Linearity and range

The mixed standard stock solution (100 μg/mL of Losartan potassium and 10 μg/mL of Metolazone) was further diluted to get Losartan potassium and Metolazone concentration in the range of 2–12 μg/mL and 0.2–1.2 μg/mL respectively. Linearity of the method was studied by injecting six concentrations of the drug prepared in the mobile phase in triplicate into the LC system keeping the injection volume constant. The peak areas were plotted against the corresponding concentrations to obtain the calibration graphs.

#### Precision

The precision of the method was verified by repeatability and intermediate precision studies. Repeatability studies were performed by analysis of three different concentrations 2, 6, 10 μg/mL for Losartan potassium and 0.2, 0.6, 1.0 μg/mL for Metolazone six times on the same day. The intermediate precision of the method was checked by repeating studies on three different days.

#### Limit of detection and limit of quantitation

Limits of detection (LOD) and quantification (LOQ) represent the concentration of the analyte that would yield signal-to-noise ratios of 3 for LOD and 10 for LOQ, respectively. To determine the LOD and LOQ, serial dilutions of mixed standard solution of Losartan potassium and Metolazone was made from the standard stock solution. The samples were injected in LC system and measured signal from the samples was compared with those of blank samples.

#### Robustness of the method

To evaluate robustness of a HPLC method, few parameters were deliberately varied. The parameters included variation of flow rate, percentage of acetonitrile in the mobile phase and solvents from different lot were taken. Robustness of the method was done at three different concentration levels 2, 6, 10 μg/mL and 0.2, 0.6, 1.0 μg/mL for Losartan potassium and Metolazone respectively.

#### Specificity

The specificity of the method towards the drug was established through study of resolution factor of the drug peak from the nearest resolving peak.The peak purity of Losartan potassium and Metolazone was determined by comparing the spectrum at three different regions of the peak i.e. peak start (S), peak apex (M) and peak end (E). Effect of excipients of formulation was studied for whether it interfered with the assay.

#### Accuracy

Accuracy of the method was determined by applying the method to drug sample (Losartan potassium and Metolazone combination tablet) to which known amount of Losartan potassium and Metolazone standard powder corresponding to 80, 100 and 120 % of label claim had been added (Standard addition method), mixed and the powder was extracted and analyzed by running chromatogram in optimized mobile phase.

### Analysis of a marketed formulation

To determine the content of Losartan potassium and Metolazone in conventional tablet (Brand name: METOZ – L 25, Batch No. 107, Label claim: 25 mg Losartan potassium and 2.5 mg Metolazone per tablet, Manufactured by: Centaur Pharmaceutical Pvt. Ltd., GOA), twenty tablets were weighed to determine the main weight and finely powdered. The weight of the tablet triturate equivalent to 25 mg of Losartan potassium and 2.5 mg Metolazone was transferred into a 50 mL volumetric flask containing 30 mL acetonitrile, sonicated for 30 min and diluted upto 50 mL with acetonitrile. The resulting solution was centrifuged at 3000 rpm for 5 min and the drug content of the supernatant was determined (500 and 50 μg/mL for Losartan potassium and Metolazone respectively). Supernatant was taken and after suitable dilution the sample solution was then filtered using 0.45-micron filter (Millipore, Milford, MA). The above stock solution was further diluted with mobile phase (acetonitrile: water in the ratio 60:40 v/v) to get sample solution of 25 and 2.5 μg/mL for Losartan potassium and Metolazone respectively. A 20 μL volume of sample solution was injected into HPLC, six times, under the conditions described above. The peak areas were measured at 237 nm and concentrations in the samples were determined using multilevel calibration developed on the same HPLC system under the same conditions using linear regression equation.

## Results and discussion

The results of validation studies on simultaneous estimation method developed for Losartan potassium and Metolazone in the current study involving acetonitrile: water (60:40, *v/v*) are given below.

### Linearity

Losartan potassium and Metolazone showed good correlation coefficient (r^2^ = 0.9993 for Losartan potassium and 0.9991 for Metolazone) in given concentration range (2–12 μg/mL for Losartan potassium and 0.2–1.2 μg/mL for Metolazone). The mean values of the correlation coefficient, slope and intercept were 0.9993 ± 0.55, 18319 ± 1.18 and 458605 ± 1.82 for Losartan potassium and 0.9991 ± 0.75, 124101 ± 1.21 and 313054 ± 1.09 for Metolazone respectively.

### Precision

The results of the repeatability and intermediate precision experiments are shown in (Table 1). The developed method was found to be precise as the RSD values for repeatability and intermediate precision studies were < 2 %, respectively as recommended by ICH guidelines.

### LOD and LOQ

Signal-to-noise ratios of 3:1 and 10:1 were obtained for the LOD and LOQ respectively. The LOD and LOQ were found to be 1 μg/mL and 2 μg/mL for Losartan potassium and 0.01 μg/mL and 0.02 μg/mL Metolazone respectively.

### Robustness of the method

Each factor selected (except columns from different manufacturers) was changed at three levels (−1, 0 and 1). One factor at the time was changed to estimate the effect. Thus, replicate injections (*n* = 6) of mixed standard solution at three concentration levels were performed under small changes of three chromatographic parameters (factors). Insignificant differences in peak areas and less variability in retention time were observed (Table 2).

### Specificity

The peak purity of Losartan potassium and Metolazone was assessed by comparing their respective spectra at the peak start, apex and peak end positions i.e., r (S, M) = 0.9989 and r (M, E) = 0.9989. A good correlation (r = 0.9997) was also obtained between the standard and sample spectra of Losartan potassium and Metolazone respectively. Also, excipients from formulation were not interfering with the assay.

### Recovery Studies

As shown from the data in Table 3, good recoveries of Losartan potassium and Metolazone in the range from 99 to 101% were obtained at various added concentrations.

### Analysis of a formulation

**Fig. 3.**
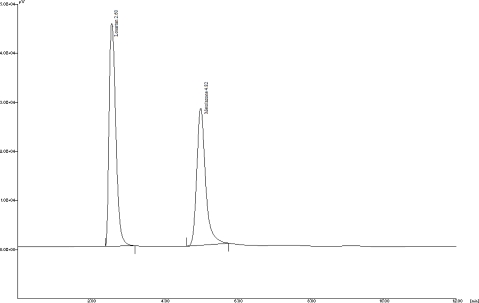
HPLC chromatogram of pharmaceutical formulation of Losartan potassium and Metolazone (20 μg/mL and 2 μg/mL)

Experimental results show that there is no interference from any of the excipients which are normally present with that of standard Losatan potassium and Metolazone (Figure 3). The drug content was found to be 99.50% for Losartan potassium and 99.40% for Metolazone. Two different lots of Losartan potassium and Metolazone combination tablets were analyzed using the proposed procedures as shown in (Table 4).

## Conclusion

A HPLC method was developed and validated as per ICH guidelines. UV detection allowed an accurate quantitation of the chromophoric compounds.

The drug was analysed by HPLC method using Thermo Hypersil BDS–C_18_ (250 mm × 4.6 mm, 5.0 μm) from Germany with isocratic conditions and simple mobile phase containing acetonitrile: water (60:40) at flow rate of 0.8 mL/min using UV detection at 237 nm. The procedure has been evaluated for the linearity, accuracy, precision and robustness in order to ascertain the suitability of the analytical method. The method was also applied to marketed samples. It has been proved that the method is selective and linear between concentration range 2–12 μg/mL for Losartan potassiumand 0.2–1.2 μg/mL for Metolazone. LOD was found to be 1 μg/mL and LOQ was found to be 2 μg/mL for Losartan potassium and LOD was found to be 0.01 μg/mL and LOQ was found to be 0.02 μg/mL for Metolazone.

Statistical analysis proves that the method is suitable for the analysis of Losartan potassium and Metolazone as bulk drug and in pharmaceutical formulation without any interference from the excipients. It may be extended to study the degradation kinetics of Losartan potassium and Metolazone and also for its estimation in plasma and other biological fluids.

## Figures and Tables

**Tab. 1.** Precision studies

**Concentration (μg/mL)**	**Repeatability (n=6)**			**Intermediate precision (n=6)**		
	**Measured conc. ±SD**	**(%) RSD**	**Recovery (%)**	**Measured conc. ±SD**	**(%)RSD**	**Recovery (%)**
**Losartan potassium**						

2	1.99± 1.10	1.62	99.50	1.98 ± 0.76	1.52	99.00
6	5.90± 1.20	0.90	98.33	5.98 ± 1.03	1.17	99.66
10	9.97 ± 1.80	0.95	99.70	10.10 ± 1.84	1.32	101.00

**Metolazone**						

0.2	0.198± 0.59	0.85	99	0.201± 0.72	0.91	100.5
0.6	0.598± 1.85	1.04	99.66	0.605± 1.77	0.76	100.83
1	0.99± 2.65	0.89	99.00	1.01± 2.34	0.66	101.00

**Tab. 2.** Robustness testing^a^ (n = 3)

**Factor^a^**	**Level**	**Retention time**	**Retention factor**	**Asymmetry**
**Losartan potassium**				

A: Flow rate (mL/min)				

0.7	−1	2.60	0.04	1.19
0.8	0	2.57	0.03	1.15
0.9	+1	2.51	0.01	1.11
Mean ± SD (n = 3)		2.56 ± 0.07	0.02 ± 0.01	1.15 ± 0.04

B: % of acetonitrile in the mobilephase (v/v)				

59	−1	2.61	0.04	1.18
60	0	2.57	0.03	1.15
61	+1	2.52	0.08	1.12
Mean ± SD (n = 3)		2.56 ± 0.05	0.05 ± 0.01	1.15 ± 0.03

C: Solvents of different lots				

First lot		2.55	0.02	1.15
Second lot		2.57	0.03	1.17
Mean ± SD (n = 3)		2.56 ± 0.01	0.02 ± 0.01	1.16 ± 0.01

**Metolazone**				

A: Flow rate (mL/min)				

0.7	−1	4.85	0.94	1.15
0.8	0	4.81	0.92	1.11
0.9	+1	4.78	0.91	1.07
Mean ± SD (n = 3)		4.81 ± 0.05	0.92 ± 0.01	1.11 ± 0.04

B: % of acetonitrile in the mobile phase (v/v)				

61	−1	4.84	0.93	1.16
60	0	4.81	0.92	1.11
86	+1	4.79	0.91	1.06
Mean ± SD (n = 3)		4.81 ± 0.06	0.92 ± 0.02	1.11 ± 0.05

C: Solvents of different lots				

First lot		4.81	0.92	1.11
Second lot		4.80	0.92	1.10
Mean ± SD (n = 3)		4.80 ± 0.01	0.92 ± 0.01	1.10 ± 0.01

a Three factors wereslightly changed at three levels (−1, 0, 1).

**Tab. 3.** Recovery studies (n = 6)

**Label claim (mg/tablet)**	**Amount added (mg)**	**Total amount (mg)**	**Amount Recovered (mg) ± % RSD**	**% Recovery**
**Losartan potassium**				

25	20 (80%)	45	44.98 ± 0.96	99.91
25	25 (100%)	50	50.10 ± 1.01	100.20
25	30 (120%)	55	55.20 ± 0.78	100.36

**Metolazone**				

2.5	2.0 (80%)	4.5	4.48 ± 1.16	99.84
2.5	2.5 (100%)	5.0	5.05± 1.40	101.00
2.5	3.0 (120%)	5.5	5.52 ± 0.98	100.36

**Tab. 4.** Analysis of commercial formulation

**Losartan potassium (25 mg)**	**Losartan potassium found (mg per tablet)**	
	**Mean ± SD (n= 6)**	**Recovery (%)**

1^st^ Lot	24.85 ± 1.06	99.40
2^nd^ Lot	24.90 ± 0.94	99.60

**Metolazone (2.5 mg)**	**Metolazone found (mg per tablet)**	
	**Mean ± SD (n= 6)**	**Recovery (%)**

1^st^ Lot	2.48 ± 1.16	99.20
2^nd^ Lot	2.49 ± 1.04	99.60
